# Developments in scalable strategies for detecting early markers of cognitive decline

**DOI:** 10.1038/s41398-022-02237-w

**Published:** 2022-11-09

**Authors:** Robert Whelan, Florentine M. Barbey, Marcia R. Cominetti, Claire M. Gillan, Anna M. Rosická

**Affiliations:** 1 School of Psychology, Trinity College Dublin, Dublin, Ireland; 2 Global Brain Health Institute, Trinity College Dublin, Dublin, Ireland; 3 Cumulus Neuroscience Ltd, Dublin, Ireland; 4 Department of Gerontology, Universidade Federal de São Carlos, São Carlos, Brazil

**Keywords:** Neuroscience, Biomarkers, Psychology

## Abstract

Effective strategies for early detection of cognitive decline, if deployed on a large scale, would have individual and societal benefits. However, current detection methods are invasive or time-consuming and therefore not suitable for longitudinal monitoring of asymptomatic individuals. For example, biological markers of neuropathology associated with cognitive decline are typically collected via cerebral spinal fluid, cognitive functioning is evaluated from face-to-face assessments by experts and brain measures are obtained using expensive, non-portable equipment. Here, we describe scalable, repeatable, relatively non-invasive and comparatively inexpensive strategies for detecting the earliest markers of cognitive decline. These approaches are characterized by simple data collection protocols conducted in locations outside the laboratory: measurements are collected passively, by the participants themselves or by non-experts. The analysis of these data is, in contrast, often performed in a centralized location using sophisticated techniques. Recent developments allow neuropathology associated with potential cognitive decline to be accurately detected from peripheral blood samples. Advances in smartphone technology facilitate unobtrusive passive measurements of speech, fine motor movement and gait, that can be used to predict cognitive decline. Specific cognitive processes can be assayed using ‘gamified’ versions of standard laboratory cognitive tasks, which keep users engaged across multiple test sessions. High quality brain data can be regularly obtained, collected at-home by users themselves, using portable electroencephalography. Although these methods have great potential for addressing an important health challenge, there are barriers to be overcome. Technical obstacles include the need for standardization and interoperability across hardware and software. Societal challenges involve ensuring equity in access to new technologies, the cost of implementation and of any follow-up care, plus ethical issues.

## Introduction

The proportion of the world’s population over the age of 60 will rapidly increase in the coming decades [1]. Cognitive decline is more likely with increasing age: this decline is primarily due to pathological processes and not age per se [2, 3]. Many neurodegenerative diseases have a long prodromal phase of several years, providing a window of opportunity to identify cognitive decline when impairment is non-existent or has little impact on daily function [4]. Existing methods for detecting cognitive decline are best suited for scenarios where symptoms have already manifested (e.g., a referral following subjective cognitive impairment) but are not appropriate when longitudinal monitoring of asymptomatic individuals is required. For example, biomarkers such as hyperphosphorylated tau protein (p-tau) and amyloid-beta (Aβ) can identify neuropathology associated with neurodegenerative diseases such as Alzheimer’s Disease (AD), prior to any cognitive decline. However, these biomarkers are typically obtained invasively from cerebrospinal fluid (CSF) via lumbar puncture. Neuropsychological tests of cognition typically require specialist administration, are insensitive to subtle declines in cognition and a patient’s performance can vary day-to-day. Structural and functional neuroimaging technologies such as magnetic resonance imaging (MRI) and positron emission tomography (PET) can detect and predict cognitive decline years before its detection via traditional neuropsychological assessment tools [5]. However, obtaining these brain data is costly and requires rigorous protocol standardization to be meaningful [6].

Scalable methods for early detection of cognitive decline would have several advantages. Even in the absence of disease-modifying therapies, which remain largely in development [7], there is a benefit to using scalable measures for screening cognitively unimpaired (CU) individuals. Early detection of cognitive decline may reduce adverse outcomes such as loss of autonomy [8] and may also mitigate the high healthcare costs that occur in the decade before formal diagnosis [9] (e.g., by providing homecare to prevent falls or infections [10] or failure to take prescribed medicine). In the event that disease-modifying therapies for neurodegenerative diseases become widely available, healthcare systems will require scalable measures for identifying patients at an early stage of the disease. A combination of a cognitive instrument and blood-based biomarker tests to triage patients at the primary care level could eliminate wait lists after the first 3 years and increase correctly identified cases by about 120,000 per year [11], primarily because referrals for PET or CSF-based biomarkers would be restricted to patients for whom disease-modifying treatment is possible (reducing annual health system expenditure by $400–700 million in the United States). With respect to clinical trials, it can be challenging to identify sufficient numbers of participants who meet the criteria for inclusion (e.g., increased brain amyloid) [12]. Improved detection could identify those at the earliest stages of the disease process, a status that could be confirmed by invasive methods. As we discuss below, a benefit of scalable measures is that clinical trial data can be recorded frequently, potentially detecting treatment effects sooner than scenarios where in-clinic assessments are collected periodically.

**Fig. 1:**
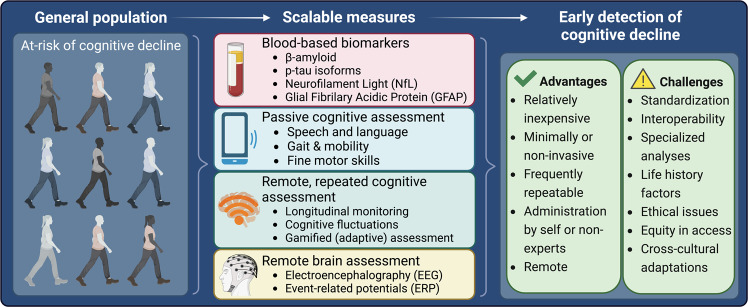
An overview of scalable measures for early detection of cognitive decline. Left panel: within the general population, those deemed “at-risk” would benefit most from early screening. Middle panel: List of scalable measures and some examples in each category. Right panel: Advantages and Challenges associated with scalable measures.

Here, we outline some emerging approaches that can provide scalable, repeatable, relatively non-invasive and comparatively inexpensive strategies for early detection of cognitive decline. These strategies are linked by two common themes. First, the data collection methods are simpler than extant clinical approaches because they do not rely on measurements obtained by professionals in dedicated settings. Rather, data are collected passively, by participants themselves or by non-experts. Second, analysis of the data *does* require highly sophisticated methods, which are designed and/or performed by specialists. These new approaches include new techniques in analysis of blood-based biomarkers that allow neuropathology to be detected from peripheral blood samples with high sensitivity and specificity. Where available, we report metrics incorporating specificity (i.e., proportion of healthy participants correctly classified) and sensitivity (i.e., the proportion of patients correctly classified). The area under the curve of the receiver operating characteristic (AROC) is a summary measure incorporating both sensitivity and specificity (AROC = 1 denotes perfect classification, .5 denotes random performance). Although AROC values such as .9 are sometimes used heuristically as a threshold for clinical tests, the desired sensitivity and specificity are situation-specific (e.g., in the context of population screening where disease prevalence is low, high specificity is desirable to avoid large numbers of false positive cases). The sample size required for any analysis also depends on the disease prevalence. When disease prevalence is low (cf. case-control designs) the sample size needs to be sufficient to accurately quantify sensitivity and specificity. Use of passive measurements collected via smartphone can unobtrusively record features relevant to cognition (collecting data almost continuously). Specific cognitive processes (e.g., memory) can be monitored regularly using ‘gamified’ cognitive tasks, potentially in combination with at-home recording of electroencephalography (EEG). Frequent repeated assessment promises richer and more reliable data than traditional snapshot assessment in the clinic [13], especially relevant because cognitive performance of older adults is more sensitive to external factors such as time of day [14, 15] or fluctuations in stress [16]. We describe the practical challenges when translating validated research methodologies into the healthcare pathway. An overview of this topic is summarized in Fig. 1.

### Peripheral-blood-based measures of neuropathology

Typically, biomarkers associated with cognitive decline are based on analysis of CSF, involving an invasive lumbar puncture and associated risks (e.g., infection). In contrast, blood-based biomarkers can be collected in a wide range of settings, such as tertiary care centers, increasing accessibility. However, detecting biomarkers in blood is challenging, not least because some biomarker levels, such as amyloid-β (Aβ), are tenfold more concentrated in CSF than in blood [17]. Blood contains several proteins, peptides, nucleic acids, lipids, metabolites, exosomes, and cellular components that present diurnal variations in their concentrations [18]. Biomarkers degrade in the liver or directly in the blood by proteases, adhere to plasma proteins or blood cells, and are excreted from the kidneys [19]. However, newer platforms can detect biomarkers present at very low concentrations in blood after having crossed the blood-brain barrier. Luminex xMAP, single-molecule array (SIMOA), immunomagnetic reduction (IMR), and immunoprecipitation mass spectrometry (IP-MS) assays, among others, are based on the principle of the Enzyme-Linked Immunoassay (ELISA) and have improved sensitivity versus conventional biomarker assays [18, 20, 21].

Previous studies related to blood-based biomarkers for detecting cognitive impairment focused on mild cognitive impairment (MCI) or AD dementia. Increasing attention is being given to evaluating blood-based biomarkers in cognitively unimpaired (CU) older adults who are at risk of developing AD, and biomarkers have predicted cognitive decline in a prospective cohort study [22]. In this section, we focus on longitudinal studies in CU participants deemed at risk (e.g., defined by Aβ status and/or genetic risk) of developing dementia.

Aβ peptides can aggregate and form oligomers and fibrils, resulting in amyloid plaque deposition, one of AD’s histopathological hallmarks [23]. Aβ peptides vary in size from 39–43 amino acids, with Aβ40 being the most abundant in CSF (about 60% of total Aβ), despite being less prone to aggregate. On the other hand, Aβ42 has a higher propensity to form toxic oligomers, which are present in CSF decades before AD onset [24, 25]. The Aβ42/40 ratio has been shown to improve diagnostic performance in routine clinical use [26] and is robust to influence from pre-analytical or analytical factors. An early study using plasma showed an Aβ42/40 ratio reduction in AD dementia versus subjective cognitive decline and MCI, with moderate accuracy (AROC = 0.68) [27]. Subsequently, other sensitive methods have been developed to quantify Aβ in blood plasma [28], and have shown Aβ42/40 ratio as a biomarker in the progression to MCI or AD dementia in CU individuals enrolled in large cohort studies [29–33]. Furthermore, the combination of plasma Aβ42/40 ratio with age and APOE-ε4 genotype could identify amyloid positivity with higher accuracy (AROC = 0.90) [30].

In AD and other tauopathies, tau undergoes post-translational modifications, resulting in aggregation and in the formation of neurofibrillary tangles [34]. Together with Aβ peptides, neurofibrillary tangles are a pathological hallmark of AD [35–37]. Different isoforms of plasma phosphorylated tau (p-tau) may be early biomarkers of cognitive decline (cf. plasma total tau) [6, 38, 39], with different assay platforms able to differentiate AD from CU participants [40, 41]. The most studied tau isoform is p-tau181, with several works reporting higher plasma levels associated with future cognitive decline over time in CU individuals [25, 42–50]. Plasma p-tau181 had superior accuracy versus CSF p-tau181 (AROC = 0.94–0.98 and AROC = 0.87–0.91, respectively) for predicting AD progression in CU individuals over time [46]. From plasma, the p-tau231 isoform differentiated persons with AD from Aβ−CU older adults and discriminated AD patients from those with non-AD neurodegenerative disorders, as well as from Aβ−MCI patients [51]. Notably, p-tau231 levels in plasma increase earlier than p-tau181 – before the threshold for Aβ PET positivity has been reached and in response to early brain tau deposition [51]. Therefore, p-tau231 may be a particularly useful biomarker of AD pathology. Plasma p-tau217 is another promising blood-based biomarker that may play a role in the spread of neocortical tangles in AD. P-tau217’s first increases in plasma are driven by Aβ aggregation and may appear largely before the spread of tau tangles outside of the medial temporal lobe [52]. Higher p-tau217 levels have been associated with steeper rates of cognitive decline, with a greater risk of converting to AD and with morphological brain alterations [1, 53–55].

Neurofilament light **(**NfL) plays a crucial role in the assembly and maintenance of the axonal cytoskeleton chain. After an axonal injury or neuronal degeneration, NfL is released into interstitial fluid and eventually into CSF and plasma [56, 57]. NfL levels are increased in frontotemporal dementia [58], small vessel disease [59], Parkinson’s disease [60] and AD [61]. The results of cross-sectional studies comparing plasma NfL concentrations and cognitive performance are mixed: some studies have found associations [62], whereas others did not [63, 64]. Longitudinal studies, however, showed that increasing levels of plasma NfL were significantly associated with declines in attention and global cognition, even after a short 15-month follow-up period [64]. In a separate study with CU participants, mean plasma levels increased 3.4 times faster in participants who subsequently developed AD than those who remained cognitively healthy [65]. In a study that investigated the NfL plasma levels in adults (mean age = 48 years) with a mean follow-up of 4.3 years, initial NfL levels were associated with a faster decline in normalized mental status scores in Whites and those >50 years old [66]. Taken together, NfL may be a predictive blood-based biomarker for global cognitive impairment.

Glial fibrillary acidic protein (GFAP) is a type-III intermediate filament component of the cytoskeleton of mature astrocytes and a marker of astroglial activation induced upon brain damage, during CNS degeneration or in the aged brain [67]. In CU individuals at risk of developing AD, GFAP predicted brain PET Aβ + (AROC = 0.76), outperforming CSF GFAP (AROC = 0.69) and other glial markers (CSF chitinase-3-like protein 1, YKL-40: AROC = 0.64; and Triggering Receptor Expressed on Myeloid Cells 2: AROC = 0.71). These results were independent of tau-PET burden, suggesting plasma GFAP is an early marker associated with brain Aβ pathology but not with tau aggregation [68]. Combining plasma GFAP with other information improved classification of Aβ+ compared to Aβ−: adding plasma GFAP plus age, sex, and APOE-ε4 carriage improved the AROC from 0.78 to 0.91 [69]. In addition, GFAP might be a prognostic biomarker to predict incident dementia. Higher baseline GFAP levels in CU participants were associated with a steeper rate of decline in memory, attention, and executive functioning [70]. In MCI participants, plasma GFAP detected AD pathology and predicted conversion to AD dementia (AROC = 0.84); in the latter case, adding APOE-ε4 or age to the model did not significantly improve the accuracy of the diagnosis [71]. However, other studies have reported a link between plasma GFAP level and progression from MCI to dementia [70].

Combining different types of blood-based biomarkers can better predict change over time [72]. Combining data from p-tau181 and NfL––but not Aβ42/Aβ40––produced the most accurate prediction (AROC = 0.88) of 4-year conversion to AD, a result that was validated in a separate cohort. A study with a long follow-up compared baseline blood-based biomarkers (Aβ misfolding, NfL, p-tau181 and GFAP) in 308 participants: 68 of whom developed dementia within 17 years. Among individual measures, Aβ misfolding was the best predictor (AROC = 0.78), followed by GFAP (0.74), NfL (0.68) and p-tau181 (0.61). However, the strongest predictor was a combination of Aβ misfolding, GFAP and APOE status (AROC = 0.83). With respect to preclinical AD, a variety of blood-based biomarkers–p-tau181, p-tau217, p-tau231, GFAP, NfL and Aβ42/40––and Aβ pathology were compared in at-risk individuals (divided into two groups, over and under 65 years old) [73]. In combination with age, sex and APOE ε4 status, the best predictors of CSF-determined Aβ status were p-tau231 (AROC = 0.81 and 0.83 for younger and older groups, respectively) and p-tau217 were (AROC = 0.76 and 0.89 for younger and older groups, respectively). Studies such as these have great potential as tools for recruitment and outcome measures in clinical trials.

### Practical considerations for blood-based biomarkers

The technology for scalable blood-based biomarkers is quite mature. For example, there is already a blood-based test for AD based on the Aβ42/40 ratio (measured by mass spectrometry), age and APOE-ε4 genotype [74], which is concordant with PET imaging scans in 94% of cases (see ref. [75]). In the context of blood-based biomarkers, a standardized operating procedure for plasma handling was produced by the Standardization of Alzheimer’s Blood Biomarkers working group [76] to describe best practices for sample pre-analytical handling (collection, preparation, dilution, and storage). These recommendations will likely bring more standardized results and, consequently, more robust comparisons among different studies evaluating blood-based biomarkers for cognitive decline. There remain practical challenges to widespread implementation of blood-based biomarkers. Once extracted, blood cells need to be separated from plasma, requiring several minutes in a centrifuge before aliquoting and freezer storage within 2 h [77]. These steps require both expertise and expensive laboratory equipment (including reliable storage at −80 °C), which are typically not available in primary healthcare settings.

### Passive assessment of cognition

Increasing smartphone and tablet usage presents new opportunities for expanding the availability and reducing the cost of cognitive assessment and for improving the precision and reducing the burden of cognitive testing. For example, in 2021, 61% of U.S. adults aged over 65 years owned a smartphone [78], an almost 5-fold increase since 2012 [79]. Various wearable and in-home sensors have been employed with the aim of detecting cognitive decline (for overviews refs. [80, 81]). However, using built-in smartphone sensors––already in the pockets of a large proportion of the population––allows passive monitoring to scale up dramatically. Extant smartphone-based research has focused on assessments of movement (e.g., gait, mobility, fine motor skills) and of language and speech problems, all of which have been associated with cognitive decline [82–85].

Mobile technologies can be used for automatic analysis of speech and language impairments that may signal cognitive decline. For example, automated speech analysis of the linguistic features captured during a tablet-administered picture description task distinguished MCI/AD patients from CU individuals [86]. Moreover, unlike standard neuropsychological test scores, one of these linguistic features–language coherence–declined significantly faster in the MCI/AD group than in CU on a 6-month follow-up, suggesting utility for monitoring language abilities over time. In a separate study, spoken answers to cognitive assessments were recorded on a tablet in order to generate a range of features (e.g., number of pauses, verbal fluency). These features were then used to develop models that differentiated among patients with subjective cognitive impairment, MCI, AD and mixed dementia patients with up to 92% accuracy [87]. Speech and language can also be assessed in less structured, naturalistic settings, such as during phone calls or typing. For example, natural language processing of speech data was passively collected during regular monitoring phone calls in a small sample of older adults with or without AD [88] and linguistic features (e.g., atypical repetition) differentiated AD from CU participants (AROC = 0.75–0.91). In addition to spoken language, features derived from touchscreen typing classified older adults with or without MCI (AROC = 0.75) [89]. Relevant features included aspects of fine motor movement (rigidity, bradykinesia, alternate finger tapping) and those related to language (e.g., lexical richness, grammatical and syntactical complexity).

Although passive measurements of communication bear an obvious relation to cognition, other features–such as gait–may also accurately predict cognitive decline. Assessing gait using smartphone tools alone is currently not specific enough for detecting AD in the general population [80]. Wearable accelerometers, however, can differentiate among dementia subtypes (AD, dementia with Lewy bodies and Parkinson’s disease) based on gait characteristics with moderate accuracy (AROC = 0.403–0.799 for the different wearable gait metrics) [90]. Real-life mobility of older adults can also be measured through smartphones using global positioning system data. Indeed, passive smartphone measures have been shown to correlate with cognitive abilities better than laboratory indicators of mobility capacity [91].

### Practical considerations for passive assessment

One of the potential benefits of passive assessment lies in utilizing the smart devices that people already possess. However, the low cost, high scalability and accessibility of such an approach has to be weighed against issues that are outside of researchers’ control. Data can be lost simply due to internet connection problems. Hardware in commercially available devices is heterogeneous (e.g., the quality of motion and acceleration sensors varies widely). Priorities of smartphone developers and researchers differ: many smart devices automatically shut down background applications to extend battery life, leading to data loss. The format of data generated from smartphone devices prioritizes user-friendly dashboards rather than a form suitable for researchers to perform quantitative analyses.

Data privacy is a major concern for passive data collection. Consent to passive monitoring of cognition via a smartphone application appears higher in participants with more technology experience and lower in healthcare professionals [92]. Worryingly, not all smartphone applications intended for neuropsychiatric conditions seem to have a privacy policy, and if this is available at all, it is often inappropriately complex for lay people [93]. It is possible, however, to effectively anonymize GPS data in order to prevent re-identification use of ancillary data [94]. Given these concerns, it is important to inform participants about what happens to their data in an accessible, transparent way.

### Remote, repeated cognitive assessment

Cognitive assessment remains the most common method for clinical diagnosis of disease-related cognitive decline, despite the recent shift towards biological biomarkers [95, 96]. However, identifying subtle changes in cognition outside the clinic – in cognitively unimpaired individuals – requires valid and reliable tools that can be administered frequently. Recent research has made strides to make cognitive assessment deliverable via desktop computer or smartphone. For example, the *Test My Brain Digital Neuropsychology Toolkit* [97] was developed and made available rapidly to meet a growing need for remote neuropsychological assessment during the COVID-19 pandemic. Tests in this toolkit probe a range of cognitive abilities including memory, processing speed and executive function: these tests all achieved ‘acceptable’ to ‘very good’ reliability despite being self-administered. Other cognitive assessment tools under development include the *Boston Remote Assessment for Neurocognitive Health* (BRANCH [98], designed to capture the first signs of cognitive decline in preclinical AD using smartphone tests. Validation work has shown that BRANCH has biological relevance, with composite BRANCH scores negatively correlated with amyloid and entorhinal tau levels [98].

Remote cognitive assessment has the benefit that it facilitates longitudinal data collection. Repeated assessment allows performance to be evaluated relative to an individual’s own baseline, which is important given that short-term fluctuations can account for up to 50% of variance on some cognitive tests across years [99]. Memory is often the earliest cognitive function to noticeably decline and is a cognitive function that is obviously suited to longitudinal assessment [100]. Smartphone assessment of memory is not only more convenient but can allow researchers to manipulate features of the design among participants to assess what works best. For example, repeated smartphone assessment was used to measure preclinical AD-related changes in long-term associative memory [100] across varied memory retention intervals of between 1–13 days. It was found that retention intervals of at least 3 days were needed to be sufficiently sensitive to differences in recall and recognition performance in adults without diagnosed cognitive impairment.

Longitudinal assessment using smartphones can also allow researchers to capture periodic fluctuations in cognition. This is important because increased variability in cognitive performance itself predicts cognitive decline in older adults, particularly on speeded [101] or selective attention tasks [102]. An assessment of the psychometric characteristics of very frequent and brief repeated smartphone assessment [103] found that cognitive test scores, averaged across 14 days of 5-times-a-day assessment, had a between-person reliability of 0.97–0.99. However, the tests still manifested sufficient within-person variability to capture cognitive fluctuations between occasions (within-person reliability of 0.41–0.53). Furthermore, smartphone-administered brief cognitive assessment repeated twice a day in multiple short sessions across 12 months could disentangle long- and short-term changes in cognitive performance [104]. Most of the variance in cognitive performance was due to between-person differences and short-term within-person fluctuations. Long-term within-person variability–the metric needed to detect cognitive decline–accounted for only approximately 14% of variance in cognition. Fluctuations in cognitive performance can differ by cognitive status. Utilizing a repeated smartphone assessment protocol, diurnal patterns in cognitive performance distinguished individuals at risk of AD from healthy older adults [15]. Specifically, time-of-day effects–lower performance in afternoon vs. morning–on an associative memory task were stronger in individuals with abnormal levels of AD biomarkers. This type of variability would be very difficult to detect using laboratory-based protocols.

**Fig. 2:**
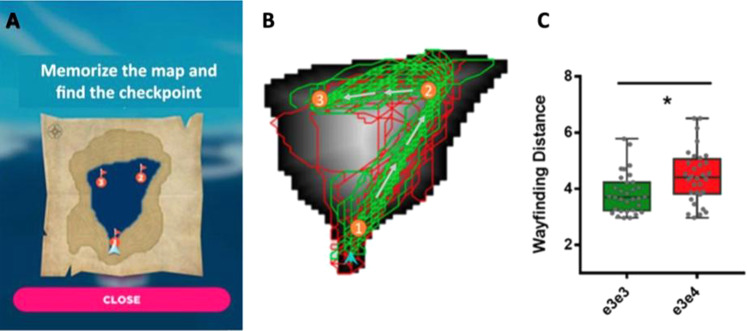
The way-finding game Sea Hero Quest is an example of a gamified smartphone tool that is sensitive to an increased genetic risk of AD (figure reprinted with permission from ref. [117]). **A** Screenshot from the game. **B** The spatial trajectories of APOE-ε4 positive (red) vs. APOE-ε4 negative (green) participants on a selected Sea Hero Quest level. **C** The way-finding distance on the game differed significantly based on APOE-ε4 status.

Although repeated cognitive assessment is desirable, cognitive tasks can be repetitive, boring, too difficult, too easy [105] and may have practice effects that affect their validity [106]. To solve these problems, there is growing interest in the use of gamification or “serious games” in cognitive assessment [107–109]. Gamified assessment aims to reduce testing anxiety [110] and increase task engagement and enjoyment without affecting performance [108]. Many studies have successfully adapted gamified assessment methods in older adults with or without cognitive impairment [111–114]. Gamified assessment can provide better construct and ecological validity than simple laboratory-based tasks thanks to a richer, more realistic context [115, 116]. Gamification is especially well suited for the assessment of navigation abilities or spatial memory because games can provide an immersive experience in 3D environments. For example, a smartphone application assessed way-finding in a high-quality 3D environment and showed that spatial navigation ability on the application was more sensitive to genetic risk for AD (APOE-ε4 status) than a classic visual episodic memory test (see Fig. 2) [117].

‘Adaptive testing’ is a common feature of gamified tasks, which is a procedure wherein tasks become more or less challenging to provide a tailored experience for the end-user. This allows cognitive tests to arrive at a reliable estimate of a person’s ability faster and more efficiently and avoids ceiling and floor effects that can harm test sensitivity. Adaptive testing also has the benefit that it promotes engagement on the part of the user and reduces frustration, making people more likely to play for longer and more frequently [118]. A driving scenario game took this approach to assess attention and executive function via tablet [119]. The game is a closed-loop system that dynamically adjusts to keep the difficulty at 80% for all players and takes approximately 7 min to complete. In a recent proof-of-concept study, the game was able to distinguish those with cognitive impairment in a multiple sclerosis (MS) population from those without [119].

### Practical considerations for remote cognitive assessment

Ideally, metrics of cognitive functioning should be *interoperable* (i.e., easily exchanged and interpreted across systems), although this can be difficult to achieve in practice. For example, differences in the technical parameters of smartphones affect the accuracy of task presentation and measurement particularly for timed tasks that involve very rapid presentation of stimuli or on tasks that become more or less difficult based on screen size [120]. These differences are not random: cognitive task performance varies systematically per device type and operating system, which is partially explained by demographic characteristics like education, age and gender, suggesting that different people access different devices [121]. Interoperability is also hindered because researchers working in this area preferentially develop new assessment tools rather than validating and generalizing existing tools [121] (cf. the Open Digital Health initiative, https://opendigitalhealth.org).

There is less experimental control with regards to distractions and identity verification for remotely collected data but this is counteracted by greater precision and standardization of the stimuli vs. pen-and-paper tasks [120, 121]. Moreover, assessment in real-life conditions can be more ecologically valid than laboratory-based single assessments in carefully controlled conditions (e.g., for tasks that examine memory retention over several days). A further concern is that tasks ideal for repeated assessment need to be short to remain engaging, which means fewer trials and therefore less reliable measures. Repeated administration of tasks can improve reliability by aggregating across multiple timepoints.

### Repeatable, remote brain assessment

Electroencephalography (EEG) is a non-invasive technique that detects the synchronous activation of cortical pyramidal neurons from the scalp. It can be used either to measure large-scale oscillatory neural population spontaneous activity during quiet wakefulness or it can be time-locked to an event. In contrast to MRI and PET, EEG technology does not involve exposure to radioactive isotopes or magnetic fields. A growing body of evidence shows that EEG is sensitive to cognitive decline, at least. As we discuss later, EEG can also potentially allow cognitive decline to be monitored via at-home recording of brain activity because the technology can be miniaturized and made portable [122].

Resting state EEG (rsEEG) may be particularly useful as a scalable method because the participant does not have to engage in a specific task, yet rsEEG appears sensitive to cognitive decline over time, albeit in groups with mild symptoms at baseline (cf. prospectively in a healthy cohort). For example, 54 MCI, 50 mild AD, and 45 CU older adults each had their EEG recorded 1-year apart [123]. At baseline, alpha-band power was lowest in the mild AD group, highest in the CU group, whereas the MCI group had intermediate values. At follow-up, the MCI group’s alpha power was further decreased, suggesting that rsEEG could be sensitive to disease progression. Similarly, in a separate study [124] there were no differences in neuropsychological test performance for participants, either Aβ positive or negative, in a 2-year follow-up. However, rsEEG, specifically the ratio of θ:α power, changed significantly over time in participants who were Aβ+. A comparison of 88 older adults with mild AD versus 35 CU across one year reported increased widespread delta power and decreased power of widespread alpha and posterior beta [125]. Furthermore, the topographies of the rsEEG power spectrum appear to be sensitive to disease stage. Differences in rsEEG power spectrum densities between mild AD patients and CU controls were the largest around temporal lobes while differences between advanced AD patients and controls were largest around frontal regions [126, 127]. A review of results from 14 studies that applied machine learning to rsEEG data reported classification accuracies between CU and MCI patients between 77–98%, sensitivity between 75–100% and specificity between 75–97% [128]. A study with a large sample (*n* = 496) healthy older adults reported that resting-state prefrontal biomarkers could predict global cognition (Mini-Mental State Examination score) with moderate accuracy (maximum intraclass correlation = 0.76) [129].

When time-locked to an event (e.g., a visual stimulus or a motor response), the electrical potentials recorded via EEG are called event-related potentials (ERPs) that can reveal – with temporal resolution in the range of milliseconds – the neural correlates of early sensory processes and of higher cognitive functions such as decision-making. The P300 ERP is often evoked using oddball tasks, during which participants should attend to the presence of an infrequent stimulus [130], and is characterized by a positive ERP deflection from approximately 300 ms after presentation of the infrequent stimulus. The P300 ERP is thought to reflect decision making and context-updating processes [131] and is particularly promising for detecting cognitive decline [126, 127, 132], with the benefit that it seems generally robust to gender and education [133] (cf. ref. [134]. P300 latency correlates with degree of cognitive deficit in AD [123, 127, 135–137]; and is increased in AD compared to MCI, and MCI patients in turn have longer latencies than age-matched controls [126, 127]. The P300 latency is correlated with cognitive impairment, as measured by the Mini-Mental State Exam [138] and the Alzheimer’s Disease Assessment Scale–Cognitive Subscale [139]. Finally, using EEG phase-amplitude coupling measures extracted from an oddball task, 15 CU were distinguished from 25 MCI with an accuracy of 95%, a sensitivity of 96%, and a specificity of 93% [140].

In contrast to the P300 ERP, which requires a participant’s attention, the mismatch negativity (MMN) ERP is produced by passive auditory oddball paradigms in which a train of frequent tones are interspersed with rare (‘deviant’) tones differing in duration or frequency [141]. The MMN ERP is thought to reflect automatic sensory processing and to act as a perceptual prediction error signal, with a latency of 100–200 ms post deviant stimulus presentation and ERP amplitude maximal at frontocentral sites [142]. The MMN ERP can distinguish MCI from AD patients [143] and amnestic MCI from healthy controls [142, 144]. MMN amplitude appears to decrease in AD for interstimulus intervals longer than 3 seconds, suggesting that sensory memory traces decay faster in AD patients compared to healthy controls [144]. Demonstrating the utility of ERPs, whereas age and education could not predict episodic memory or attention/executive functions at 5-year follow-up, MMN metrics explained an additional 36% of the variance in episodic memory performance [144] at follow-up.

Given that laboratory-based EEG appears suitable for detecting cognitive decline, scalability can be achieved by recording EEG outside of the laboratory (i.e., remotely). Furthermore, remote EEG can be conducted by unsupervised non-expert users [122, 145–149]. Remote EEG platforms vary on a range of parameters such as the electrode type (i.e., requiring a conductive medium or not), number and placement of electrodes; portability; user-friendliness and whether or not the signals are transmitted wirelessly [122]. Precise synchronization between the EEG recording device and the presentation of exogenous stimuli can be achieved by pairing the EEG headset to a handheld tablet [147, 148] or laptops [147].

**Fig. 3:**
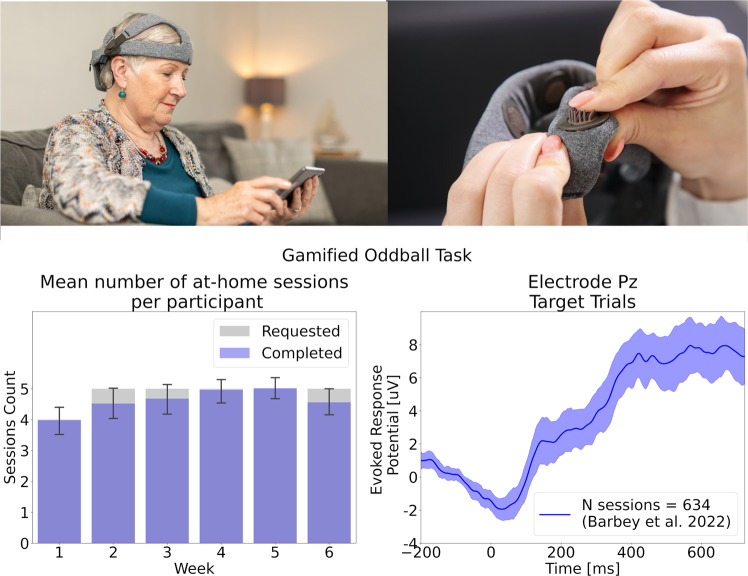
Example of a portable, dry EEG platform. Upper left panel: The platform (Cumulus Neuroscience) consists of a wireless 16-channel dry sensor EEG headset linked to an Android tablet for task presentation. Upper right panel: Flexible Ag/AgCl coated dry EEG electrodes. Lower left panel: Figure adapted with permission from ref. [155] showing weekly adherence of a cohort of 50 healthy older adults (+55 years old) with a 6-week at-home EEG recording protocol. Lower Right Panel: Figure adapted with permission from ref. [153] showing averaged event-related potentials from target trials extracted from a gamified Oddball task, collected remotely by participants themselves.

The nomenclature for remote EEG reflects the variety of possible configurations. ‘Portable’ refers to systems designed for use outside the laboratory. ‘Mobile’ refers specifically to technology that can be used in motion, such as walking [149]. ‘Wet’ and ‘Dry’ refer to the type of electrodes used to conduct the signal from the scalp: the former requiring a conductive medium (electrolytic gel or water), the latter relying on mechanical pressure against the scalp to ensure contact. In order to ensure standardized electrode placement on the scalp, rigid headsets are preferred for non-expert use [149]. Portable, dry EEG devices can yield data that are similar in quality to those obtained by wet laboratory-based EEG systems [150–153] with comparable ERP amplitudes and latencies between wet and dry EEG [154], significant positive correlations (*r* = 0.54–0.89) between wet and dry EEG recordings for both spectral components and ERPs [155], and intra-class correlations between 0.76–0.85 across three separate testing time points using dry EEG [156]. Figure 3 shows an example of a portable EEG system and associated remotely collected data.

Most usability studies of portable EEG have been conducted in laboratories and/or have been overseen by trained technicians [149, 151, 155, 157]. Limited data are available regarding the usability of such technology when participants self-administer EEG longitudinally from the home. However, one study [148] reported data from 89 CU adults (age range 40–80 years) asked to complete at-home recordings with a portable wireless dry EEG platform 5 times per week for 3 months. Each session was approximately 30 mins and consisted of gamified versions of tasks commonly administered under EEG–two-stimulus oddball, flanker, delayed match-to-sample and N-back–plus rsEEG. Participants were not compensated, yet mean adherence was 4.1 sessions per week with a low attrition rate of 11/89 participants (neither adherence nor attrition were related to age). A high percentage of recordings (96% of 3,603 sessions) contained usable EEG data. These results suggest that it is feasible for participants to collect longitudinal brain data from the home, which is essential if EEG is to be used for detecting or monitoring cognitive decline

### Practical considerations for remote EEG assessment

Integrating measures of cognitive decline into a healthcare system requires standardized metrics, yet subtle differences in task design (e.g., frequency of stimulus presentation) can change the nature of EEG results [144]. Unlike psychometric tests, there are no established norms for cognitive ERP amplitude and latency although the development of standardized metrics may address this issue [158, 159]. Self-administered EEG may not be feasible for those with motor impairment (e.g., Parkinson’s disease). Putting on the headset and adjusting electrodes requires some manual dexterity: some assistance may be needed for these participants. Finally, many remote technologies often rely on a good WiFi connection to download tasks and upload recorded data.

### Pathway to healthcare practice: opportunities and challenges

The approaches described above–peripheral blood-based markers associated with neuropathology, passive monitoring of cognition and remote brain assessment–offer potential strategies for detecting cognitive decline. However, deploying these strategies at scale in diverse real-world healthcare settings is subject to several considerations: scientific, societal and ethical. In this section, we discuss these challenges and some potential solutions.

Early diagnosis raises complex ethical issues, including the right (not) to know and communication challenges regarding the probabilistic nature of any assessment [160]. Although smartphone and EEG tools described here accurately measure cognitive function, they are not yet comparable with clinical tools. In particular, the specificity–the proportion of negative cases correctly identified–needs to be high before a tool for identifying cognitive decline is clinically useful. Identifying cases with low or no symptoms may cause individual distress and unduly burden health systems by creating the ‘worried well’, particularly when there are limited treatment options for diseases that cause cognitive decline [161]. An economic burden may result from the cost of technologies, such as dry EEG and plasma biomarker platforms, although these should reduce if manufactured at scale. Many methods have been developed for Western populations: for example, between 2000–2019, 41% of studies using automated speech and language processing for AD monitoring were conducted in English, with other studies focusing mostly on Western-European languages [84]. It will be important to ensure these approaches are suitable for low- and middle-income countries, particularly because cognitive decline is becoming a bigger issue in those regions [162, 163]. At a more fundamental level, some methods lack an evidence base per se. A review of 83 available smartphone apps related to the most disabling neuropsychiatric conditions found that only 18% seemed evidence-based [93].

An important stepping-stone to real-world implementation is deployment of scalable methods and tools in large, prospective cohorts of CU participants. Indeed, some of these studies are already underway. For example, the Early Detection of Neurodegenerative diseases (EDoN) [164] project aims to collect data from passive sensors and easily obtained clinical measures to detect the earliest signatures of dementia. EDoN’s ultimate goal is to develop a digital toolkit to deployed at a population level for people over age 40. Notably, at first, a range of digital metrics will be recorded. Subsequently, a data-driven approach (machine learning) will identify a subset of measures that, in combination, are most predictive. Combining a variety of tools in this way may improve specificity. A Swedish-based prospective study – BioFINDER (Biomarkers For Identifying Neurodegenerative Disorders Early and Reliably; https://biofinder.se/)–seeks to validate blood-based biomarkers for the diagnosis of AD and Parkinson’s Disease in primary care settings. Participants in BioFINDER complete a wide range of specialized, gold-standard measures (e.g., neuroimaging), against which scalable methods can be compared.

## Conclusion

In this review, we described approaches for detecting cognitive decline at scale, each of which varied in their development status. Improvements in standardization and interoperability are needed for tools designed for assaying specific cognitive functions to be widely deployed. However, in the near future, blood plasma measures of neuropathology, passive smartphone data collection and resting-state EEG could plausibly be implemented at scale. It may be possible to provide a direct-to-consumer test using p-tau as a blood-based biomarker to identify those most at risk of cognitive decline [165]. Alternatively, or in addition, cognitive functioning can be detected using passive “digital biomarkers” to detect early signs of disease [166]. The methods described here can also be used to identify participants at-risk of cognitive impairment for inclusion in clinical trials, to monitor progression of cognitive decline and to assay treatment responses over time. For clinical trials, the ability to monitor cognitive and brain responses frequently (perhaps even on a daily basis) would allow researchers to map the evolution of any treatment response over time, and potentially to identify individual differences associated with effective medication response. Obtaining behavior and brain data outside the laboratory may radically change approaches for detecting cognitive decline.
